# Characterization of Respiratory Symptoms Among Youth Using Heated Tobacco Products in Hong Kong

**DOI:** 10.1001/jamanetworkopen.2021.17055

**Published:** 2021-07-14

**Authors:** Lijun Wang, Jianjiu Chen, Lok Tung Leung, Zhi-Ming Mai, Sai Yin Ho, Tai Hing Lam, Man Ping Wang

**Affiliations:** 1 School of Public Health, University of Hong Kong, Hong Kong, China; 2 Department of Epidemiology, Mailman School of Public Health, Columbia University, New York, New York; 3 School of Nursing, University of Hong Kong, Hong Kong, China

## Abstract

**Importance:**

Heated tobacco products (HTPs) are promoted as less harmful than combustible cigarettes but epidemiological evidence is scarce, especially in youth.

**Objective:**

To investigate the associations of persistent respiratory symptoms with HTP use, cigarette use, and dual use among Hong Kong youth.

**Design, Setting, and Participants:**

This was a territorywide cross-sectional school-based survey conducted from October 2018 to July 2019 using an anonymous questionnaire. Schools were randomly invited from a proportionate stratified sample in all 18 districts of Hong Kong. Poisson regression models using generalized estimating equations yielded adjusted prevalence ratios (APRs) of respiratory symptoms in (1) former and current HTP (vs never) users in the whole sample and stratified by cigarette use status and (2) exclusive HTP and dual users vs exclusive cigarette users. Statistical analysis was performed from October 2020 to March 2021.

**Exposures:**

Former and current use of cigarettes, HTPs, e-cigarettes, and other tobacco products.

**Main Outcomes and Measures:**

Respiratory symptoms for 3 consecutive months in the past 12 months.

**Results:**

The study included 33 627 students with a mean (SD) age of 14.8 (1.9) years; 51.3% (18 171) were boys. Respiratory symptoms were reported by 16.3% (n = 5549) of all students, 29.3% (n = 226) of current users of e-cigarettes, 31.2% (n = 314) of current users of cigarettes, and 33.5% (n = 179) of current users of HTPs. Respiratory symptoms were associated with former (APR, 1.30; 95% CI, 1.06-1.59) and current (APR, 1.59; 95% CI, 1.23-2.06) vs never HTP use and current vs never cigarette use (APR, 1.50; 95% CI, 1.30-1.74) after adjusting for various tobacco use. Associations between respiratory symptoms and current vs never HTP use were observed in never (APR, 1.88; 95% CI, 1.36-2.59) and former (APR, 2.15; 95% CI, 1.12-4.12) cigarette users, but not in current cigarette users (APR, 1.24; 95% CI, 0.97-1.59). Respiratory symptoms were associated with exclusive ever HTP use (APR, 1.46, 95% CI, 1.15-1.86) and ever dual use (APR, 1.29; 95% CI, 1.08-1.54) vs exclusive ever cigarette use. There was no association between exclusive current HTP (vs cigarette) use and respiratory symptoms (1.40; 95% CI, 0.93-2.11).

**Conclusions and Relevance:**

This cross-sectional study found that former and current HTP use were associated with persistent respiratory symptoms among youth, especially among never and former cigarette users. Respiratory symptoms were more prevalent in ever exclusive HTP users and ever dual users than ever exclusive cigarette users. These findings suggest that using HTPs instead of cigarettes may not reduce health risks.

## Key Points

**Question:**

Is heated tobacco product (HTP) use associated with fewer respiratory symptoms when compared with cigarette smoking in youth?

**Findings:**

In this cross-sectional study with 33 627 youths from 88 secondary schools, persistent respiratory symptoms were more prevalent in HTP users than nonusers, especially among never and former cigarette smokers. Persistent respiratory symptoms were associated with exclusive ever HTP use and ever dual use vs exclusive ever cigarette smoking, and comparable in current users of HTPs and cigarettes.

**Meaning:**

Youth HTP and cigarette use were similarly associated with respiratory symptoms, suggesting that both HTP and cigarette use should be prevented in youth.

## Teaser

This cross-sectional study investigates associations of persistent respiratory symptoms with heated tobacco product use, cigarette use, and dual use among youth in Hong Kong.

## Introduction

Heated tobacco products (HTPs) heat processed tobacco to generate an aerosol for inhalation and have been marketed as less harmful than combustible cigarettes. Studies have shown that exposure to harmful and potentially harmful chemicals can be significantly reduced if smokers switch completely from combustible cigarettes to HTPs.^1^ However, whether reduced exposure would result in reduced harms to human health has not been confirmed.

The potential for harm reduction by instead using HTPs is controversial. Laboratory tests showed that nicotine and total particulate matter in mainstream HTP aerosol were comparable to cigarette smoke.^2^ HTPs generated chemicals that were present in cigarette smoke, although at lower levels, but also chemicals that were absent in cigarette smoke, such as glycerol and the lethal formaldehyde cyanohydrin.^3,4,5^ Experiments on rats conducted by the tobacco industry have shown that exposure to HTP aerosol was associated with substantial pulmonary inflammation and immunomodulation.^6^ In addition, rats exposed to HTP aerosol had similar impairments of vascular endothelial function, and their serum nicotine levels (immediately after exposure) were 3.5-fold higher than those exposed to cigarette smoke (70.3 [SD, 26.3] ng/mL in the HTP group and 15.0 [SD, 7.7] ng/mL in the cigarette group).^7^

Evidence on the health effects of HTP use in humans is limited. Glantz^8^ conducted a secondary analysis with the trial results submitted by the industry, and found no differences in most of the biomarkers of potential harm (23 of 24 in US adults, 10 of 13 in Japanese adults) between cigarette smokers and those who switched to HTPs. Moazed et al^6^ also analyzed these documents and found no improvements in pulmonary inflammation or lung function in cigarette smokers who switched to HTPs. Another trial in 50 males by Pataka et al^9^ showed significant decrease in oximetry (oxygen saturation), and increase in exhaled carbon monoxide and airway resistance immediately after HTP use in both cigarette smokers and nonsmokers. In an online survey of 102 former or current smoking adults who used HTPs, sore throat (n = 5), cough (n = 3), and headache (n = 3) were reported after using HTPs.^10^ Another online survey of 8784 Japanese individuals aged 15 to 73 years showed that asthma attack and chest pain were associated with exposure to secondhand smoke of HTPs (vs cigarettes) in the past year.^11^ Recently, 3 acute eosinophilic pneumonia cases have been linked to HTP use—2 male youths aged 16 and 20 years old,^12,13^ and a woman aged 47 years who switched from cigarettes to HTPs.^14^ By searching “(heated tobacco product OR heat-not-burn) AND (child OR adolescent)” in PubMed and Web of Science until May 8, 2021, we only found 2 studies,^15,16^ both based on the Korea Youth Risk Behavior Survey in 2018, with statistical test results, showing that ever use of HTPs was associated with current asthma (adjusted odds ratio [AOR], 3.8; 95% CI, 1.5-9.6) in never users of cigarettes and e-cigarettes, but not with current allergic rhinitis.

The tobacco industry has sought to launch HTPs in Hong Kong with a much lower tax than cigarettes, but no HTPs have been licensed to be sold (as tobacco products).^17,18^ However, untaxed HTPs are available in online and physical stores;^18^ among Hong Kong youth, 2.3% had ever used HTPs and 0.5% currently used HTPs in 2019.^19^ As youth are more vulnerable to air pollutants and tobacco smoke than adults,^20^ youth HTP use is a great public health concern. Therefore, we aimed to investigate the associations between HTP use and respiratory symptoms considering other tobacco use in Hong Kong youth.

## Methods

### Study Design and Participants

The School-Based Survey on Smoking is the largest territorywide biennial smoking survey in secondary school students (US grades 7 to 12) in Hong Kong. The present round was conducted from October 2018 to July 2019. Details of the survey methods have been reported.^21,22^ Briefly, schools were invited from a stratified random sample in all 18 districts in Hong Kong, in proportion to the total number of schools in each district. Each participating school was compensated with a book coupon worth HK $500 (US $65). Parental consent was sought before the survey. All the parents from participating schools received an invitation letter via students, and declining parents were to ask students to return a blank answer sheet during the survey. Students’ participation was voluntary even with parental consent. Students answered a paper-and-pencil questionnaire in classrooms within one class session. At least one trained research assistant per grade was available to coordinate and answer students’ enquiries. To encourage candid reporting, teachers avoided patrolling near students, and a separate anonymous answer sheet was used. Completed answer sheets were immediately sealed in front of the students and collected by research assistants. In total, 34 063 students in 88 schools returned the answer sheets, with a response rate of 94% at student-level and 23% at school-level. Given that most students were ethnic Hong Kong/Cantonese but born in different places (eg, Hong Kong, mainland China, Macau), we asked their place of birth instead of race/ethnicity. Ethics approval of this survey was obtained from the institutional review board of the University of Hong Kong/Hospital Authority Hong Kong West Cluster. This study followed the Strengthening the Reporting of Observational Studies in Epidemiology (STROBE) reporting guideline for cross-sectional studies.^23^

### Tobacco Use Measures

In the beginning of the questionnaire, we provided a brief introduction to each tobacco product: electronic cigarettes refer to electronic devices that heat a chemical solution to produce an aerosol; heated tobacco products refer to electronic devices that heat up tobacco sticks to produce an aerosol. Then the use of combustible cigarettes (CCs), HTPs, e-cigarettes and other tobacco products (eg, waterpipe, cigar, snus, etc.) were separately assessed by “Please choose one sentence which suits you most regarding the product” (options: “I have never used it,” “I have used it once or a few times,” “I used to use it occasionally, but have quit now,” “I used to use it every day, but have quit now,” “I use it occasionally,” or “I use it every day”). Ever use was defined as any use or trying of the product,^24^ and thus students who chose “I have never used it” were classified as never users, and otherwise as ever users. Current use of each product was assessed by “On how many of the past 30 days did you use the product?” (options: 0, 1-2, 3-5, 6-9, 10-19, 20-29, or 30 days). Ever users who used the product for at least 1 day in the past 30 days was classified as current users, and otherwise as former users.^24^

### Outcome Ascertainment

We asked whether students had frequent cough or phlegm for 3 consecutive months in the past 12 months (options: yes or no). Daily respiratory symptoms such as cough, congestion, or phlegm, for at least 3 months in a row are indicative of chronic bronchitis in children.^25^ These persistent symptoms have been associated with environmental pollutants and cigarette smoking, which can reflect pulmonary health and predict chronic respiratory diseases.^26,27,28^ Therefore, we used them as a simple and practical indicator of short-term health impacts of HTP use.^22^

### Covariates

We also assessed exposure to secondhand smoke at home by “On how many of the past 7 days has someone used smoking products near you at home?” (options: 0, 1, 2, 3, 4, 5, 6, or 7 days) and alcohol drinking by “Did you drink any alcoholic beverages in the past 12 months?” (options: yes or no). Sociodemographic characteristics included sex, age, grade, and perceived family affluence.^29,30^

### Statistical Analysis

We calculated the proportions of respiratory symptoms by sociodemographic characteristics, secondhand smoke exposure at home, and alcohol drinking, weighted by sex, age, and grade distribution of the underlying population provided by the Education Bureau of the Hong Kong Special Administrative Region Government. As other tobacco use is linked to both HTP use and respiratory symptoms,^28,31^ we examined the associations between HTP use and respiratory symptoms in 3 ways: (1) associations between HTP use (former and current vs never) and respiratory symptoms, adjusting for other tobacco use; (2) associations between HTP use (former and current vs never) and respiratory symptoms by CC use status (never, former, and current), adjusting for other tobacco use; and (3) associations of exclusive HTP use and dual use with respiratory symptoms compared with exclusive CC use, separately for ever and current use, and adjusting for other tobacco use. In association analyses, Poisson regression models were fitted and robust standard errors were calculated using generalized estimation equations accounting for school clustering effects^32,33^ with R version 4.0.0 (R Project for Statistical Computing) package gee (version 4.13-20). We also adjusted for the background characteristics which were associated with respiratory symptoms, as they were common confounders in the association between tobacco use and health, including sex, perceived family affluence, number of days exposed to secondhand smoke at home in the past 7 days, and alcohol drinking in the past 12 months.^34,35,36^ The present analysis excluded 72 students (0.2% of 34 063) with higher than 50% missing items and 364 students (1.1% of 34 063) with missing data on respiratory symptoms, leaving a total of 33 627 students included in the study. Missing rates were less than 0.3% for all other variables used in the present analyses.

All statistical tests were 2-sided with *P* < .05 indicating statistical significance. Statistical analysis was performed from October 2020 to March 2021.

## Results

The study sample of 33 627 students had a mean (SD) age of 14.8 (1.9) years; 18 171 (51.3%) were boys. Respiratory symptoms were reported by 16.3% of the students (n = 5549), and the proportions were higher in boys (3253 [17.8%]), the least-affluent families (1736 [18.6%]), students with more mean (SD) days of exposure to secondhand smoke at home (1.6 [2.7] days vs 1.4 [2.6] days), and alcohol drinkers (2868 [18.1%]) (*P* values < .001), but did not differ by age or grade (Table 1).

**Table 1.** Background Characteristics and Respiratory Symptoms of the Sample

Characteristic	All respondents, No. (%)^a^ (N = 33 627)	Students with respiratory symptoms, No. (%)^a^ (n = 5549)	*P* value^b^
Sex			
Boys	18 171 (51.3)	3253 (17.8)	<.001
Girls	15 456 (48.7)	2296 (14.6)	
Age, mean (SD), y	14.8 (1.9)	14.8 (1.9)	.71
Grade, secondary			
1	6206 (17.9)	1024 (16.5)	.74
2	6563 (17.1)	1082 (16.6)	
3	6666 (16.6)	1056 (15.6)	
4	6197 (16.4)	1068 (17.0)	
5	5527 (16.1)	922 (16.3)	
6	2468 (15.9)	397 (15.6)	
Perceived family affluence			
Relatively poor	9132 (27.1)	1736 (18.6)	<.001
Average	19 611 (58.7)	3000 (15.2)	
Relatively rich	4779 (14.2)	791 (16.5)	
SHS exposure, mean (SD), d^c^	1.4 (2.6)	1.6 (2.7)	<.001
Alcohol drinking	15 460 (47.0)	2868 (18.1)	<.001

Abbreviation: SHS, secondhand smoke.

^a^ Proportions unless otherwise stated, weighted by sex, age, and grade distribution of the underlying population provided by the Education Bureau of the Hong Kong SAR Government.

^b^ *P* values were differences of respiratory symptoms by sex, perceived family affluence, and alcohol drinking from χ^2^ test, and linear trends of respiratory symptoms by age, grade, and days of secondhand smoke exposure.

^c^ Mean (SD) days of secondhand smoke exposure at home in the past 7 days in all respondents and in those who experienced respiratory symptoms.

Respiratory symptoms were reported by 31.2% (n = 314) of current users of cigarettes, 33.5% (n = 179) of current users of HTPs, and 29.3% (n = 226) of current users of e-cigarettes. Models 1 and 2 in Table 2 show that respiratory symptoms were associated with former and current use of any tobacco products. After mutually adjusting for use of each product (model 3), respiratory symptoms were associated with former (adjusted prevalence ratio [APR], 1.30; 95% CI, 1.06–1.59) and current (APR, 1.59; 95% CI, 1.23–2.06) (vs never) HTP use (*P* value for trend < .001), and with current (vs never) cigarette use (APR, 1.50; 95% CI, 1.30–1.74). No interactions of tobacco use with secondhand smoke exposure or sex were found in the associations. Former and current use of e-cigarettes were not independently associated with respiratory symptoms after adjusting for use of cigarettes and HTPs. Therefore, we only classified the sample by cigarette and HTP use in the following analyses, and included use of e-cigarettes and other tobacco products as a covariate in the fully adjusted models.

**Table 2.** Associations of Respiratory Symptoms With Various Tobacco Product Use

Product	Students with respiratory symptoms, No. (%)	Model 1, PR (95% CI)^a^	Model 2, PR (95% CI)^b^	Model 3, PR (95% CI)^c^
CC				
Never	4812 (15.8)	1 [Reference]	1 [Reference]	1 [Reference]
Former	416 (19.8)	1.26 (1.14-1.38)	1.11 (1.01-1.23)	1.07 (0.96-1.20)
Current	314 (31.2)	1.98 (1.78-2.19)	1.75 (1.58-1.94)	1.50 (1.30-1.74)
*P* for trend	NA	<.001	<.001	<.001
HTP				
Never	5255 (16.1)	1 [Reference]	1 [Reference]	1 [Reference]
Former	107 (27.9)	1.74 (1.46-2.07)	1.52 (1.28-1.80)	1.30 (1.06-1.59)
Current	179 (33.5)	2.08 (1.80-2.41)	1.87 (1.61-2.17)	1.59 (1.23-2.06)
*P* for trend	NA	<.001	<.001	<.001
e-Cigarettes				
Never	4946 (16.0)	1 [Reference]	1 [Reference]	1 [Reference]
Former	369 (20.2)	1.26 (1.14-1.40)	1.13 (1.02-1.25)	0.98 (0.88-1.10)
Current	226 (29.3)	1.84 (1.58-2.13)	1.63 (1.40-1.88)	0.96 (0.78-1.19)
*P* for trend	NA	<.001	<.001	.75
Other tobacco products				
Never	5181 (16.1)	1 [Reference]	1 [Reference]	1 [Reference]
Former	176 (24.4)	1.52 (1.30-1.77)	1.33 (1.14-1.55)	1.09 (0.91-1.30)
Current	182 (30.0)	1.86 (1.59-2.18)	1.67 (1.43-1.95)	0.86 (0.66-1.12)
*P* for trend	NA	<.001	<.001	.84

Abbreviations: CC, combustible cigarette; HTP, heated tobacco product; NA, not applicable; PR, prevalence ratio.

^a^ Adjusted for school clustering.

^b^ Adjusted for model 1 variable, sex, perceived family affluence (categorical), days of secondhand smoke exposure, and alcohol drinking. No interactions between secondhand smoke exposure and use of CCs, e-cigarettes, HTPs, or other tobacco products. No interactions between sex and use of CCs, e-cigarettes, HTPs, or other tobacco products.

^c^ Adjusted for model 2 variables and use of CCs, e-cigarettes, HTPs, and other tobacco products (never, former, and current). No interactions between secondhand smoke exposure and use of CCs, e-cigarettes, HTPs, or other tobacco products. No interactions between sex and use of CCs, e-cigarettes, HTPs, or other tobacco products.

Table 3 shows that, in never CC users, former (APR, 1.69; 95% CI, 1.30-2.20) and current (APR, 1.88, 1.36-2.59) HTP use were associated with respiratory symptoms compared with never HTP use. In former CC users, current (vs never) HTP use was associated with respiratory symptoms (APR, 2.15; 95% CI, 1.12-4.12), but there was no association for former (vs never) HTP use (APR, 1.29; 95% CI, 0.95-1.75) (*P* = .10). In current CC users, the APRs of respiratory symptoms in former (APR, 1.10; 95% CI, 0.78-1.54) and current (APR, 1.24; 95% CI, 0.97-1.59) (v never) HTP users were not statistically significant. The APRs of respiratory symptoms increased with HTP use status (never, former and current) among never and former CC users (*P* values for trends ≤ .01), but showed no marked trend among current CC users (*P* for trend = .21). When using never users of both products as the reference, any use of HTPs or cigarettes was associated with respiratory symptoms (eTable in the Supplement).

**Table 3.** Associations Between HTP Use and Respiratory Symptoms by Cigarette Use

CC	HTP	Students with respiratory symptoms, No. (%)	Model 1, PR (95% CI)^a^	Model 2, PR (95% CI)^b^	Model 3, PR (95% CI)^c^
Never	Never	4755 (15.7)	1 [Reference]	1 [Reference]	1 [Reference]
	Former	30 (29.1)	1.86 (1.43-2.41)	1.67 (1.29-2.15)	1.69 (1.30-2.20)
	Current	27 (33.8)	2.15 (1.66-2.78)	1.93 (1.47-2.53)	1.88 (1.36-2.59)
	*P* for trend	NA	<.001	<.001	<.001
Former	Never	362 (19.1)	1 [Reference]	1 [Reference]	1 [Reference]
	Former	39 (23.9)	1.25 (0.94-1.67)	1.20 (0.91-1.59)	1.29 (0.95-1.75)
	Current	13 (36.1)	1.89 (1.11-3.22)	1.92 (1.12-3.28)	2.15 (1.12-4.12)
	*P* for trend	NA	.008	.01	.008
Current	Never	142 (29.2)	1 [Reference]	1 [Reference]	1 [Reference]
	Former	38 (31.4)	1.09 (0.78-1.52)	1.08 (0.76-1.55)	1.10 (0.78-1.54)
	Current	153 (32.8)	1.13 (0.93-1.37)	1.22 (1.00-1.50)	1.24 (0.97-1.59)
	*P* for trend	NA	.13	.22	.21

Abbreviations: CC, combustible cigarette; HTP, heated tobacco product; NA, not applicable; PR, prevalence ratio.

^a^ Adjusted for school clustering.

^b^ Adjusted for model 1 variable, sex, perceived family affluence (categorical), days of secondhand smoke exposure, and alcohol drinking. No interactions between secondhand smoke exposure and use of CCs or HTPs. No interactions between sex and use of CCs or HTPs.

^c^ Adjusted for model 2 variables and use of CCs, HTPs, and other tobacco products (eg, e-cigarettes) (never, former, and current). No interactions between secondhand smoke exposure and use of CCs, HTPs, or other tobacco products. No interactions between sex and use of CCs, HTPs, or other tobacco products.

Table 4 shows that respiratory symptoms were associated with exclusive ever HTP use (APR, 1.46; 95% CI, 1.15-1.86) and ever dual use (APR, 1.29; 95% CI, 1.08-1.54) vs exclusive ever CC use. As regards current use, the APRs were not statistically significant for exclusive current HTP use (APR, 1.40; 95% CI, 0.93-2.11) and current dual use (APR, 1.19; 95% CI, 0.94-1.49) compared with exclusive current CC use.

**Table 4.** Respiratory Symptoms in Exclusive and Dual Users of Cigarettes and HTPs

Variable	Students with respiratory symptoms, No. (%)	Model 1, PR (95% CI)^a^	Model 2, PR (95% CI)^b^	Model 3, PR (95% CI)^c^
Ever use status				
Exclusive CC use	507 (21.3)	1 [Reference]	1 [Reference]	1 [Reference]
Exclusive HTP use	47 (32.2)	1.52 (1.21-1.91)	1.52 (1.21-1.91)	1.46 (1.15-1.86)
Dual use	217 (31.0)	1.46 (1.24-1.71)	1.44 (1.24-1.68)	1.29 (1.08-1.54)
Current use status				
Exclusive CC use	180 (29.7)	1 [Reference]	1 [Reference]	1 [Reference]
Exclusive HTP use	26 (38.8)	1.31 (0.90-1.91)	1.37 (0.92-2.04)	1.40 (0.93-2.11)
Dual use	153 (32.8)	1.11 (0.93-1.32)	1.20 (1.00-1.44)	1.19 (0.94-1.49)

Abbreviations: CC, combustible cigarette; HTP, heated tobacco product; PR, prevalence ratio.

^a^ Adjusted for school clustering.

^b^ Adjusted for model 1 variable, sex, perceived family affluence (categorical), days of secondhand smoke exposure, and alcohol drinking. No interactions between secondhand smoke exposure and use of CCs or HTPs. No interactions between sex and use of CCs or HTPs.

^c^ Adjusted for model 2 variables and use of CCs, HTPs, and other tobacco products (including e-cigarettes) (never, former, and current). No interactions between secondhand smoke exposure and use of CCs, HTPs, or other tobacco products. No interactions between sex and use of CCs, HTPs, or other tobacco products.

## Discussion

To our knowledge, this cross-sectional study provided the first evidence that former and current HTP use were associated with respiratory symptoms in youth, especially in never cigarette users. In former cigarette users, respiratory symptoms were associated with current HTP use, but were not associated with former HTP use. We also compared the prevalence of respiratory symptoms between HTP use and cigarette use, and found that exclusive ever HTP users and ever dual users of both products had even higher APRs of respiratory symptoms than exclusive ever cigarette users. HTP use in Hong Kong appeared later than many Western countries, and young users might have shorter use history on average, but using persistent respiratory symptoms as the outcome has allowed us to detect its health hazards. Only the Korea Youth Risk Behavior Survey in 2018 reported the health impacts of HTP use in youth,^15,16^ showing that ever HTP use (never cigarette and e-cigarette use, regardless of other tobacco use) was associated with past-year asthma, but not with past-year allergic rhinitis.

We found former and current HTP use were associated with respiratory symptoms after adjusting for use of cigarettes, e-cigarettes, and other tobacco products. In never cigarette users, the prevalence of respiratory symptoms in former and current HTP users increased by 69% and 88% compared with never HTP users. In former cigarette users, current HTP use was still associated with higher risks of respiratory symptoms, suggesting that switching from combustible cigarette smoking to HTP use did not eliminate the adverse health outcomes. In current cigarette users, the risks of respiratory symptoms for HTP use were not significantly greater, suggesting that HTP use did not provide protection for current smokers who dually used HTPs and combustible cigarettes. These results were consistent with those of a 90-day trial conducted by the tobacco industry showing that switching to HTPs did not change pulmonary inflammation or function in cigarette smokers.^6^ Both the International Tobacco Control Survey in Japanese adults and our previous study in Hong Kong youth showed that HTPs may reinforce nicotine dependence rather than serving as a cessation aid, because cigarette consumption did not vary between exclusive smokers and those who partially switched to HTPs.^37,38^

The APR of respiratory symptoms for exclusive ever HTP use was even larger than that for exclusive ever cigarette use. A similar pattern was reported in the Korean youth study: the AOR of past-year asthma was 3.59 (95% CI, 1.47-8.78) in exclusive HTP users and 1.30 (95% CI, 1.08-1.56) in exclusive cigarette users.^16^ The potential health hazards of youth HTP use in real-world conditions could be drastically underestimated if they were to be labeled as modified risk tobacco products.

Lower concentrations of toxicants do not necessarily lead to less risk. First, due to less harshness than combustible cigarettes and kid-friendly flavors, HTPs can be easier to initiate and more appealing to youth who do not use or lightly use nicotine, and facilitate persistent and frequent use.^39,40,41^ Second, despite no flames, the I Quit Original Smoking (IQOS) tobacco product sticks (Philip Morris International), with similar ingredients to cigarettes, are always charred because of pyrolysis and generate volatile and semi-volatile toxicants as in cigarette smoke, challenging the claim of “heat but not burn.”^4^ Third, harmful constituents are increasingly confirmed in HTPs. Many harmful and potentially harmful substances in the aerosol of IQOS are more than 50% higher than cigarette smoke, such as glycerol, α,β-unsaturated carbonyl compounds, 1,2-dicarbonyl compounds, furans and epoxides.^3,5^ HTPs also contain highly toxic formaldehyde cyanohydrin that is absent in cigarette smoke.^4^ Finally, HTP use would have detrimental effects similar to cigarette smoking. Evidence has shown that HTP aerosol impairs human airway cell homeostasis by increasing oxidative stress, inflammation and airway remodeling, which plays a key role in many chronic respiratory diseases caused by cigarette smoke.^42^ Despite scarce evidence from population-based studies, potential adverse health effects of HTP use have been shown by in vitro and in vivo experiments, case reports and a few small-scale human trials.^6,7,8,9,12,13,14^

The outbreak of e-cigarette or vaping product use-associated lung injury (EVALI) in the US was linked to inhaled Vitamin E acetate and/or tetrahydrocannabinol,^43^ and the pathology of the 3 HTP use-related acute eosinophilic pneumonia cases remain undetermined,^12,13,14^ alerting that we know little about the hazards of new tobacco products, especially their long-term effects. Our results in Hong Kong youth found that the health risks of e-cigarette use were mainly associated with dual use of cigarettes and HTPs, but use of new tobacco products mimics the hand-to-month smoking behaviors, which can renormalize tobacco use and act as a gateway to cigarette smoking.^44^ The Hong Kong Special Administrative Region Government proposed a total ban of e-cigarettes and HTPs in February 2019, but the bills encountered strong opposition from the tobacco industry and had not been approved as of May 8, 2021. Governmental approval of HTPs may encourage the tobacco industry to market their HTPs all over the world, despite the statement that these products are not safe. Although more research is needed to understand the short- and long-term health effects of HTPs, prompt actions should be taken to prevent further HTP use in youth, and a total ban would be the most effective solution.

### Limitations

This study had some limitations. First, self-reported tobacco use was subject to social desirability bias, although the anonymous questionnaire, separate answer sheet, and other procedures to instill confidence should encourage candid reporting.^21,22^ The associations could be overestimated due to recall bias if students with persistent respiratory symptoms were more likely to recall tobacco use, but such bias would occur similarly for different tobacco products and have little effect on the comparison between HTP and cigarette use. Second, higher risks of HTP use than cigarette use could be explained by reverse causality if cigarette users who experienced severe respiratory symptoms switched to HTPs. However, reverse causality cannot explain the associations between HTP use and respiratory symptoms in never cigarette users. Third, the low response rate at school level (23%) could lead to selection bias, although nonparticipation of schools was usually because of difficulties in arrangements rather than smoking-related issues. Finally, although respiratory symptom is a useful and sensitive outcome for even short-term HTP use in youth, more serious harms may only manifest themselves after long-term use.

## Conclusions

To our knowledge, this study is the first to report that former and current HTP use in youth were associated with respiratory symptoms, especially in never cigarette users. In former cigarette users, respiratory symptoms were associated with current HTP use. In current cigarette users, concurrent use of HTP was not associated with respiratory symptoms. Respiratory symptoms were more prevalent in ever exclusive HTP users and ever dual users than ever exclusive cigarette users. The prevalence of respiratory symptoms in exclusive current HTP users and exclusive current cigarette users was comparable. Using HTPs instead of cigarettes may not reduce health risks. Our results lend support to banning HTPs to protect youth health.

## Notes

Supplement. **eTable.** Associations of Respiratory Symptoms With Cigarette And HTP Use vs Never Use of Both Products
