# Supplementary material for: Characterization of Respiratory Symptoms Among Youth Using Heated Tobacco Products in Hong Kong
Source: JAMA Netw Open. 2021 Jul 14;4(7):e2117055. doi: 10.1001/jamanetworkopen.2021.17055 (PMC8280960; doi:10.1001/jamanetworkopen.2021.17055)
Supplement: Supplement. — eTable. Associations of Respiratory Symptoms With Cigarette And HTP Use vs Never Use of Both Products [file jamanetwopen-e2117055-s001.pdf]

Supplemental Online Content

Wang L, Chen J, Leung LT, et al. Characterization of respiratory symptoms among youth using heated tobacco products in Hong Kong. *JAMA Netw Open*. 2021;4(7):e2117055. doi:10.1001/jamanetworkopen.2021.17055

eTable. Associations of Respiratory Symptoms With Cigarette And HTP Use vs Never Use of Both Products

This supplemental material has been provided by the authors to give readers additional information about their work.

© 2021 Wang L et al. *JAMA Network Open.*

**eTable. Associations of respiratory symptoms with cigarette and HTP use vs never use of both products (n=33,627)**					
		**Respiratory symptoms**	**Model 1**	**Model 2**	**Model 3**
**CC**	**HTP**	**n (%)**	**PR (95% CI)[a]**	**PR (95% CI)[b]**	**PR (95% CI)[c]**
**Never**	**Never**	4,755 (15.7)	1	1	1
	**Former**	30 (29.1)	1.86 (1.43, 2.41)***	1.67 (1.29, 2.15)***	1.69 (1.30, 2.20)***
	**Current**	27 (33.8)	2.15 (1.66, 2.78)***	1.93 (1.47, 2.53)***	1.88 (1.36, 2.59)***
	P for trend	-	<0.001	<0.001	<0.001
**Former**	**Never**	362 (19.1)	1.22 (1.11, 1.33)***	1.08 (0.98, 1.19)	1.11 (1.00, 1.22)
	**Former**	39 (23.9)	1.52 (1.13, 2.03)**	1.30 (0.97, 1.73)	1.39 (1.03, 1.89)*
	**Current**	13 (36.1)	2.24 (1.30, 3.87)**	2.06 (1.19, 3.54)*	2.12 (1.15, 3.92)*
	P for trend	-	<0.001	<0.01	<0.01
**Current**	**Never**	142 (29.2)	1.84 (1.61, 2.11)***	1.63 (1.43, 1.87)***	1.68 (1.44, 1.96)***
	**Former**	38 (31.4)	2.05 (1.52, 2.77)***	1.81 (1.34, 2.45)***	1.88 (1.36, 2.61)***
	**Current**	153 (32.8)	2.12 (1.80, 2.49)***	1.90 (1.62, 2.23)***	1.98 (1.57, 2.50)***
	P for trend	-	<0.001	<0.001	<0.001

*P<0.05, **P<0.01, ***P<0.001. Abbreviations: PR, prevalence ratio; CI, confidence interval; CC, combustible cigarette; HTP, heated tobacco product.

[a] Adjusted for school clustering.

[b] Adjusted for Model 1 variable, sex, perceived family affluence (categorical), days of secondhand smoke exposure and alcohol drinking. No interactions between secondhand smoke exposure and use of CCs/HTPs. No interactions between sex and use of CCs/HTPs.

[c] Adjusted for Model 2 variables and use of CCs, HTPs and other tobacco products (including e-cigarettes, etc.) (never, former and current). No interactions between secondhand smoke exposure and use of CCs/HTPs/other tobacco products. No interactions between sex and use of CCs/HTPs/other tobacco products.

© 2021 Wang L et al. *JAMA Network Open.*
